# Ancestral Lineage of SARS-CoV-2 Is More Stable in Human Biological Fluids than Alpha, Beta, and Omicron Variants of Concern

**DOI:** 10.1128/spectrum.03301-22

**Published:** 2023-01-23

**Authors:** Taeyong Kwon, Natasha N. Gaudreault, David A. Meekins, Chester D. McDowell, Konner Cool, Juergen A. Richt

**Affiliations:** a Department of Diagnostic Medicine/Pathobiology, College of Veterinary Medicine, Kansas State University, Manhattan, Kansas, USA; University of Georgia

**Keywords:** SARS-CoV-2, human biological fluids, stability, variants of concern

## Abstract

SARS-CoV-2 is a zoonotic virus first identified in 2019, and has quickly spread worldwide. The virus is primarily transmitted through respiratory droplets from infected persons; however, the virus-laden excretions can contaminate surfaces which can serve as a potential source of infection. Since the beginning of the pandemic, SARS-CoV-2 has continued to evolve and accumulate mutations throughout its genome leading to the emergence of variants of concern (VOCs) which exhibit increased fitness, transmissibility, and/or virulence. However, the stability of SARS-CoV-2 VOCs in biological fluids has not been thoroughly investigated. The aim of this study was to determine and compare the stability of different SARS-CoV-2 strains in human biological fluids. Here, we demonstrate that the ancestral strain of the Wuhan-like lineage A was more stable than the Alpha VOC B.1.1.7, and the Beta VOC B.1.351 strains in human liquid nasal mucus and sputum. In contrast, there was no difference in stability among the three strains in dried biological fluids. Furthermore, we also show that the Omicron VOC B.1.1.529 strain was less stable than the ancestral Wuhan-like strain in liquid nasal mucus. These studies provide insight into the effect of the molecular evolution of SARS-CoV-2 on environmental virus stability, which is important information for the development of countermeasures against SARS-CoV-2.

**IMPORTANCE** Genetic evolution of SARS-CoV-2 leads to the continuous emergence of novel virus variants, posing a significant concern to global public health. Five of these variants have been classified to date into variants of concern (VOCs); Alpha, Beta, Gamma, Delta, and Omicron. Previous studies investigated the stability of SARS-CoV-2 under various conditions, but there is a gap of knowledge on the survival of SARS-CoV-2 VOCs in human biological fluids which are clinically relevant. Here, we present evidence that Alpha, Beta, and Omicron VOCs were less stable than the ancestral Wuhan-like strain in human biological fluids. Our findings highlight the potential risk of contaminated human biological fluids in SARS-CoV-2 transmission and contribute to the development of countermeasures against SARS-CoV-2.

## INTRODUCTION

Severe acute respiratory syndrome coronavirus 2 (SARS-CoV-2) is a recently emerged respiratory virus and the causative agent for the current pandemic. SARS-CoV-2 belongs to the genus *Betacoronavirus*, family *Coronaviridae*, order *Nidovirales*; it is an enveloped virus containing a positive-sense, single-stranded RNA genome of approximately 30 kb in length. The first two thirds of the genome (5′ to 3′) are comprised of two overlapping open reading frames (ORF), ORF1a and ORF1b, which encode two large polyproteins through a programmed −1 ribosomal frameshift (1). These polyproteins are proteolytically cleaved into a total of 16 nonstructural proteins, which are involved in virus replication and transcription as well as innate immune evasion by suppressing host factors in various signaling pathways (2). The last third of the genome encodes four structural proteins: spike (S), envelope (E), membrane (M), and nucleocapsid (N), and eight accessory proteins: ORF3a, ORF3b, ORF6, ORF7a, ORF7b, ORF8, ORF9a, and ORF9b. The trimeric S protein of SARS-CoV-2 mediates viral attachment to the host cell receptor, human angiotensin-converting enzyme 2 (ACE2), and subsequent virus-cell fusion and entry into target cells (3). The E and M proteins are components of the viral envelope and play a role in viral assembly and budding of SARS-CoV-2 (4–6). The N is an RNA-binding protein responsible for viral genome packaging (7).

SARS-CoV-2 has continued to evolve and has accumulated mutations throughout its genome since its emergence in late 2019. Due to the error-prone nature of RNA-dependent RNA polymerase, random mutations are introduced into the genome of SARS-CoV-2 during viral replication such that SARS-CoV-2 populations exist as a quasispecies. Selection pressures drive the viral population to maintain beneficial mutations which could potentially increase viral fitness. This process of mutation and selection guides the evolution of SARS-CoV-2 and contributes to the emergence and spread of new variants that can pose an increased risk to global public health. Five variants of SARS-CoV-2 have so far been designated as variants of concern (VOCs): Alpha (B.1.1.7), Beta (B.1.351), Gamma (P.1), Delta (B.1.617.2), and Omicron (B.1.1.529) (see https://www.who.int/en/activities/tracking-SARS-CoV-2-variants). The Alpha VOC was first identified in the United Kingdom in September 2020 and became the dominant strain circulating in many parts of the world until May 2021 (8). The Beta and Gamma VOCs were first identified in South Africa and Brazil, respectively; they have been responsible for small proportions of COVID-19 cases worldwide, but their emergence and continued spread have been highlighted due to their ability to evade pre-existing immunity and therapeutics (9, 10). After being first identified in October 2020 in India, the Delta VOC has spread to many other countries and replaced the previously prevalent Alpha VOC to become the dominant strain of SARS-CoV-2 worldwide (11). On November 26, 2021, the variant B.1.1.529 was designated as the Omicron VOC, which is a highly divergent variant harboring a high number of mutations, especially in the S protein. In addition, many other variants with specific genetic makers which are predicted to affect virus characteristics have been classified as variants of interest (VOIs).

To date, antigenic and virological aspects of SARS-CoV-2 VOCs, such as transmissibility, disease severity, vaccine and therapeutic efficacy and immune evasion, have been widely investigated. Most studies have explored the stability of several strains of SARS-CoV-2 on surfaces (12–14) and a few have investigated the stability of SARS-CoV-2 in human biological fluids (15–17). These studies enable us to determine the potential of SARS-Cov-2 transmission by fomites. However, the stability of different VOCs in human biological fluids has not been compared side-by-side. Studies evaluating the stability of new variant strains of SARS-CoV-2 in biological fluids is important for assessing the risk of potential fomite transmissions. Therefore, we evaluated the stability of four different SARS-CoV-2 strains, (i) an ancestral lineage A strain, (ii) an Alpha VOC, (iii) a Beta VOC, and (iv) an Omicron BA.1 VOC in human biological fluids under different environmental conditions.

## RESULTS

To characterize the detailed genetics of the virus stocks used in this study, we performed next-generation sequencing (NGS) using an Illumina Nextseq platform. The results showed that the consensus sequence of the WA-1 ancestral lineage A strain was 100% identical with the reference sequence available in GISAID (accession ID: EPI_ISL_404895) except for a synonymous mutation from C to T at position 1912. The virus stock of the Alpha VOC was 100% homologous with the GISAID reference sequence (accession ID: EPI_ISL_683466), and had several amino acid substitutions in the S protein compared to the WA-1 strain: H69del, V70del, Y145del, N501Y, A570D, D614G, P681H, T716I, S982A, and D1118H (Table S1). We also found an amino acid substitution from aspartic acid to leucine at position 3 of the nucleocapsid protein (D3L). The virus stock of the Beta VOC was homologous with the GISAID reference sequence (accession ID: EPI_ISL_678615) and contained several amino acid substitutions in the S protein compared to the WA-1 strain: L18F, D80A, D215G, L242del, A243del, L242del, K417N, E484K, N501Y, D614G, and A701V. Two additional substitutions, Q677H and R682W, were found in the S protein of the Beta VOC virus stock compared to the reference sequence. Also, proline at position 71 of the E protein was replaced with leucine in the Beta VOC (P71L) and a P252L substitution was found in NSP5 and the substitution R115L in ORF8. The consensus nucleotide sequence of the Omicron VOC stock had 100% identity to the reference sequence deposited in GISAID (accession ID: EPI_ISL_7908052). In the Omicron VOC, a total 31 amino acid substitutions, six deletions, and three insertions were found in the S protein as well as several substitutions and deletions in three other structural proteins, compared to the WA-1 strain.

**FIG 1:**
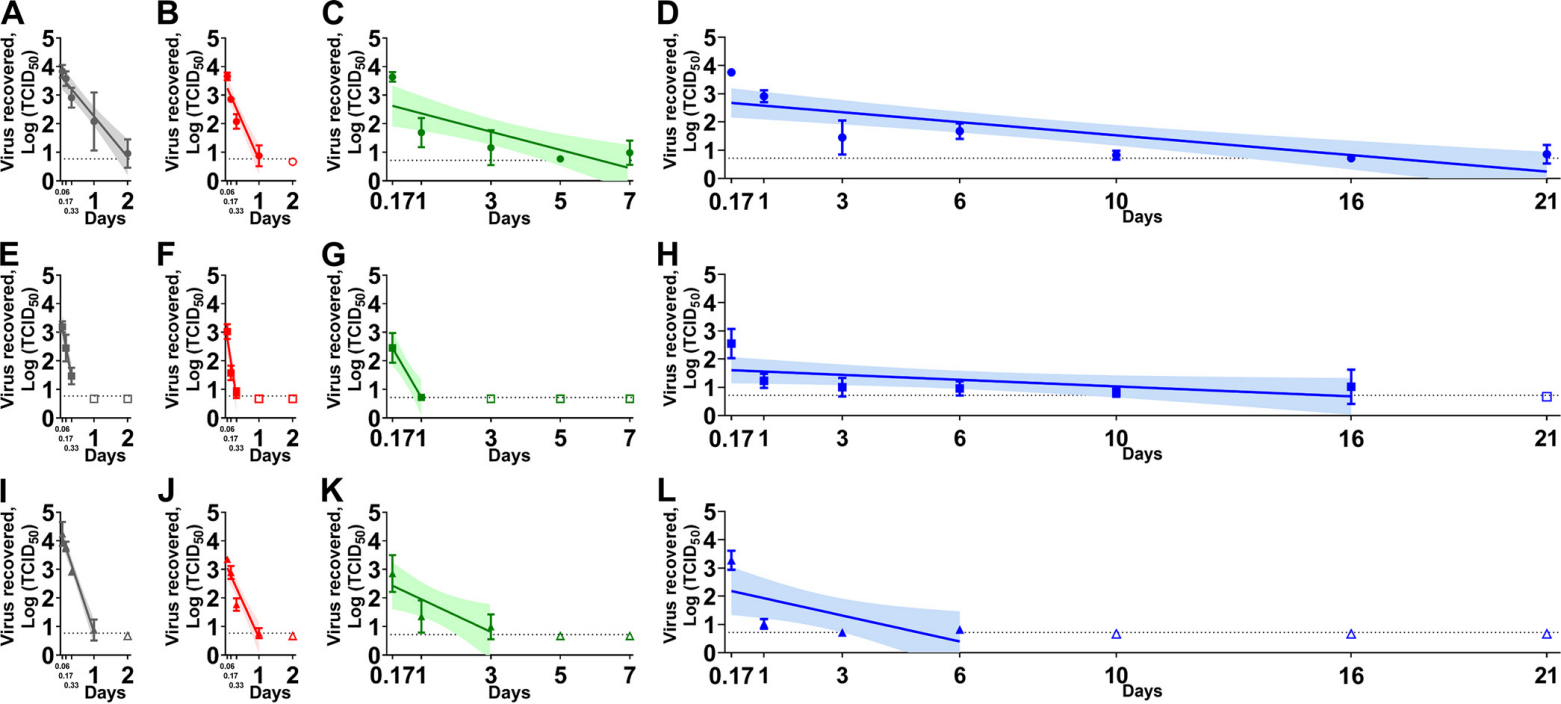
The stability of the severe acute respiratory syndrome coronavirus 2 (SARS-CoV-2) ancestral lineage A strain (A to D), the Alpha variants of concern (VOC) (E to H), and the Beta VOC (I to L) in human nasal mucus under indoor (A, E, and I), summer (B, F, and J), spring/fall (C, G, and K), and winter (D, H, and L) conditions. The cell culture derived virus (5 × 10^4^ TCID_50_) was mixed with nasal mucus in a 2 mL sealed tube and placed in a temperature and humidity-controlled chamber. After the incubation under each environmental condition, the sample was diluted in the 2 mL medium, filtered through 0.45 μm syringe filter, and titrated on Vero-TMPRSS2 cells. Virus titers were log-transformed to estimate a simple linear regression model. Virus titer at each time point was expressed as a geometric mean of three replicates and the standard deviation. A best-fit line and its 95% confidence interval of each regression model are represented by a solid line and its shaded area. The dashed line indicates the limit of detection where at least one sample out of the three replicates was positive by virus isolation, and the empty symbols represent negative samples in all three replicates. On the *x* axis, 0.06, 0.17, and 0.33 days are equal to 1.5 h, 4 h, and 8 h, respectively.

In liquid nasal mucus (Fig. 1), the WA-1 strain was more stable than the Alpha VOC under indoor, summer, and spring/fall conditions (*P* < 0.0001). Similarly, the Beta VOC survived longer than the Alpha VOC under indoor (*P* < 0.001), summer (*P* < 0.0001), and spring/fall (*P* < 0.0001) conditions. In addition, we found a significant difference in the viral decay rate between the WA-1 strain and the Beta VOC under indoor conditions (*P* < 0.01). In winter conditions, the WA-1 strain and the Alpha VOC survived significantly longer than the Beta VOC (*P* < 0.05 for WA-1 versus Beta VOC; and *P* < 0.01 for Alpha VOC versus Beta VOC).

**FIG 2:**
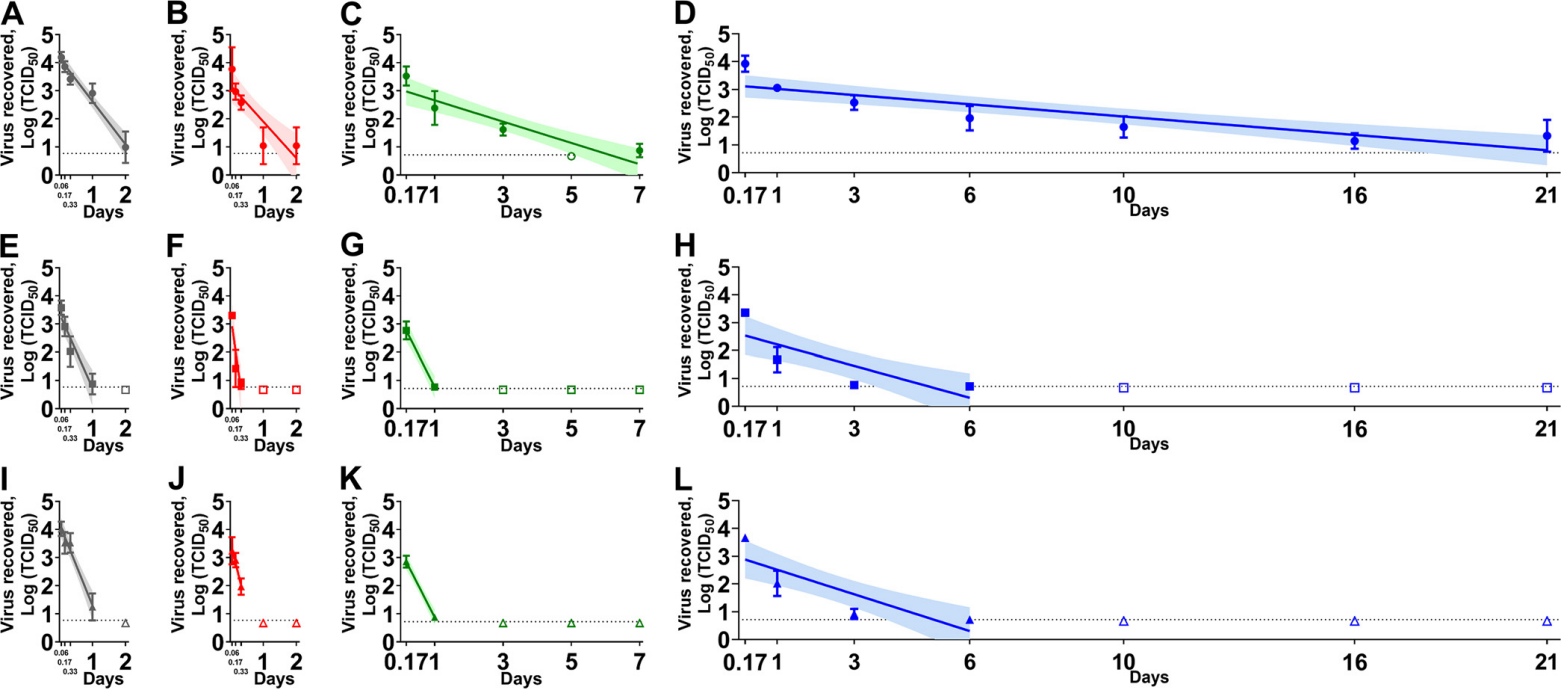
The stability of the severe acute respiratory syndrome coronavirus 2 (SARS-CoV-2) ancestral lineage A strain (A to D), the Alpha variants of concern (VOC) (E to H), and the Beta VOC (I to L) in human sputum under indoor (A, E, and I), summer (B, F, and J), spring/fall (C, G, and K), and winter (D, H, and L) conditions. The cell culture derived virus (5 × 10^4^ TCID_50_) was mixed with sputum in a 2 mL sealed tube and placed in a temperature and humidity-controlled chamber. After the incubation under each environmental condition, the sample was diluted in the 2 mL medium, filtered through 0.45 μm syringe filter, and titrated on Vero-TMPRSS2 cells. Virus titers were log-transformed to estimate a simple linear regression model. Virus titer at each time point was expressed as a geometric mean of three replicates and the standard deviation. A best-fit line and its 95% confidence interval of each regression model are represented by a solid line and its shaded area. The dashed line indicates the limit of detection where at least one sample out of the three replicates was positive by virus isolation and the empty symbols represent negative samples in all three replicates. On the *x* axis, 0.06, 0.17, and 0.33 days are equal to 1.5 h, 4 h, and 8 h, respectively.

In liquid sputum (Fig. 2), the WA-1 strain survived significantly longer than the Alpha VOC under indoor (*P* < 0.05), summer (*P* < 0.0001), spring/fall (*P* < 0.0001), and winter (*P* < 0.01) conditions. In addition, the WA-1 strain was also more stable than the Beta VOC in liquid sputum under indoor (*P* < 0.01), summer (*P* < 0.05), spring/fall (*P* < 0.0001), and winter (*P* < 0.01) conditions. Overall, the Alpha and Beta VOCs had similar stability in sputum under all conditions tested.

In liquid saliva (Table 1), the WA-1 strain and the Alpha VOC were more stable than the Beta VOC under winter conditions (*P* < 0.001 for WA-1 versus Beta VOC; and *P* < 0.01 for Alpha VOC versus Beta VOC). In liquid medium (Table 1), the half-life values of WA-1 were significantly higher than those of both, the Alpha and Beta VOCs under winter conditions (*P* < 0.05 for WA-1 versus Alpha VOC; and *P* < 0.001 for WA-1 versus Beta VOC). However, no difference in half-life values among the three strains was found in medium, nasal mucus, sputum, and saliva dried on the stainless steel surface (Table 1).

**TABLE 1:** Half-life values of the SARS-CoV-2 ancestral lineage A strain and two variants of concern in liquid biological fluids or dried on surface under indoor, summer, spring/fall, and winter conditions

Biological fluid	Virus strain	Indoor		Summer		Spring/fall		Winter	
		Liquid	Surface	Liquid	Surface	Liquid	Surface	Liquid	Surface
Nasal mucus	WA-1 lineage A	5.06 (3.93 to 7.09)^*a*^	4.07 (2.98 to 6.41)	2.7 (2.13 to 3.69)	5.21 (3.59 to 9.48)	22.65 (14.6 to 50.54)	41.9 (25.32 to 121.58)	61.88 (44.31 to 102.5)	79.62 (60.22 to 117.5)
	Alpha VOC B.1.1.7	1.14 (0.85 to 1.76)	6.55 (4.46 to 12.36)	0.95 (0.7 to 1.49)	Not determined^*b*^	3.47 (2.33 to 6.78)	49.32 (26.13 to 437.04)	123.27 (63.18 to 2506.49)	76.91 (39 to 2764.28)
	Beta VOC B.1.351	2.05 (1.78 to 2.43)	3.89 (2.61 to 7.62)	2.78 (2.18 to 3.86)	3.38 (2.65 to 4.65)	12.64 (6.91 to 74.16)	29.57 (20.29 to 54.51)	23.57 (12.85 to 141.66)	78.35 (57.32 to 123.78)
Sputum	WA-1 lineage A	4.69 (3.99 to 5.69)	11.49 (8.66 to 17.09)	5.53 (3.83 to 9.99)	5.34 (4.37 to 6.88)	19.05 (14.43 to 28.05)	36.87 (27.9 to 54.42)	65.44 (49.49 to 96.58)	87.08 (66.09 to 127.61)
	Alpha VOC B.1.1.7	2.73 (2.07 to 4.04)	11.66 (8.89 to 16.96)	0.85 (0.56 to 1.76)	7.77 (5.68 to 12.32)	3 (2.38 to 4.06)	40.81 (25.28 to 105.81)	18.92 (12.24 to 41.65)	87.74 (65.03 to 134.75)
	Beta VOC B.1.351	2.48 (2.01 to 3.24)	10.91 (8.14 to 16.55)	1.5 (1 to 3)	6.36 (5.47 to 7.59)	3.04 (2.56 to 3.73)	36.73 (24.59 to 72.47)	16.36 (11.19 to 30.43)	65.31 (51.87 to 88.15)
Saliva	WA-1 lineage A	15.34 (12.23 to 20.55)	13.9 (9.08 to 29.63)	11.1 (8.68 to 15.41)	4.84 (3.66 to 7.15)	35.62 (29.69 to 44.56)	31.92 (23.01 to 52.02)	96.11 (67.15 to 169.02)	49.81 (43.46 to 58.33)
	Alpha VOC B.1.1.7	14.09 (11.4 to 18.43)	12.49 (10.16 to 16.21)	8.87 (7.55 to 10.76)	5.7 (4.73 to 7.19)	28.56 (19.61 to 52.43)	27.37 (20.52 to 41.03)	84.04 (66.12 to 115.29)	54.14 (48.13 to 61.89)
	Beta VOC B.1.351	13.61 (10.77 to 18.49)	14.25 (11.12 to 19.83)	12.52 (9.5 to 18.36)	6.45 (4.74 to 10.09)	23.44 (20.49 to 27.37)	33.24 (28.32 to 40.23)	49.82 (44.1 to 57.24)	60.99 (47.26 to 85.96)
Medium	WA-1 lineage A	18.23 (14.73 to 23.91)	8.56 (7.76 to 9.55)	13.66 (10.84 to 18.46)	4.16 (3.56 to 4.99)	57.35 (40.76 to 96.67)	23.37 (18.4 to 32)	181.56 (138.92 to 261.77)	116.09 (85.4 to 181.23)
	Alpha VOC B.1.1.7	12.83 (10.83 to 15.73)	10.46 (9 to 12.5)	9.58 (8.07 to 11.79)	5.16 (2.84 to 27.82)	40.28 (32.01 to 54.29)	22.28 (18.57 to 27.85)	105.92 (84.46 to 141.93)	99.58 (75.9 to 144.73)
	Beta VOC B.1.351	16.23 (12.89 to 21.89)	9.21 (8.05 to 10.76)	10.86 (8.86 to 14.03)	4.92 (4.14 to 6.06)	37.13 (25.34 to 69.51)	28.16 (22.1 to 38.82)	84.75 (69.04 to 109.7)	87.97 (75.71 to 104.96)

a Half-life in hours (95% confidence interval).

b Half-life value was not calculated because the slope of simple linear regression was not significantly different from zero.

**FIG 3:**
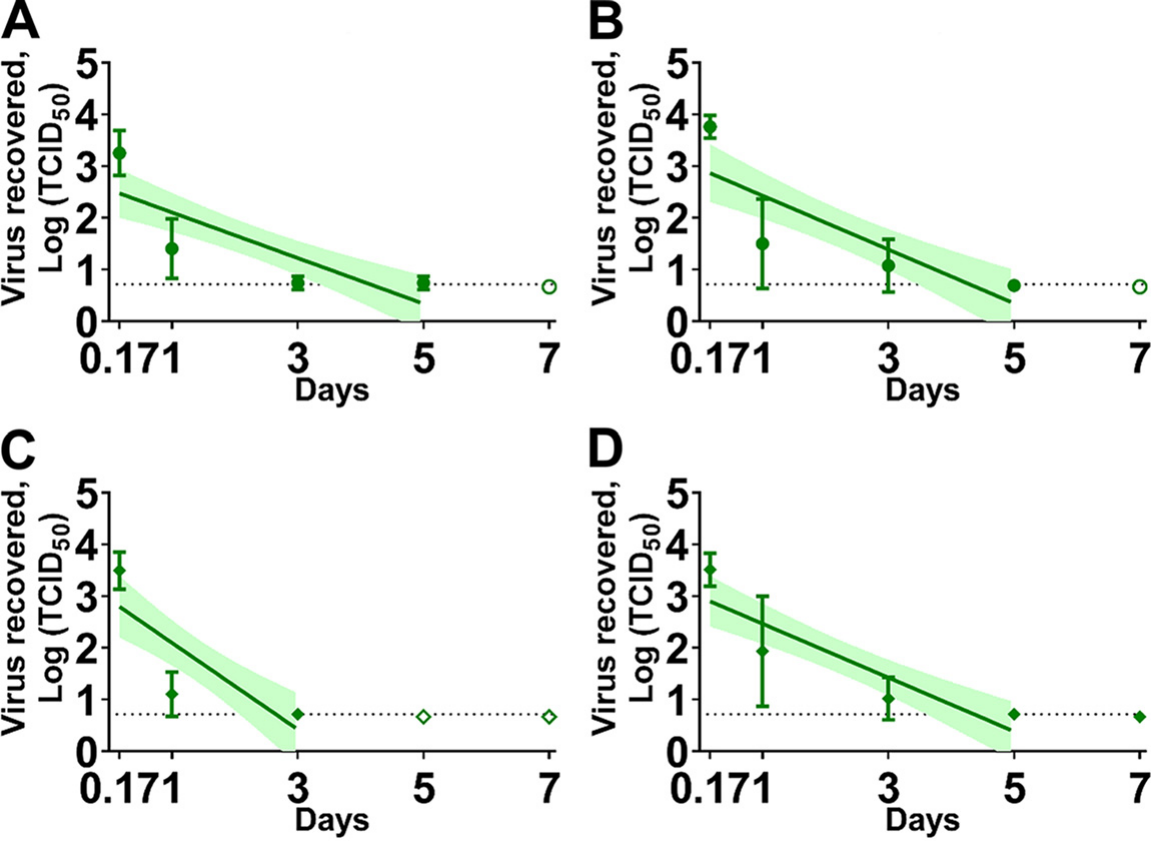
The stability of the severe acute respiratory syndrome coronavirus 2 (SARS-CoV-2) ancestral lineage A strain (A and B) and the Omicron variants of concern (VOC) (C and D) in human nasal mucus (A and C) and sputum (B and D) spring/fall conditions. The cell culture derived virus (3.1 × 10^4^ TCID_50_) was mixed with nasal mucus or sputum in a 2 mL sealed tube and placed in a temperature and humidity-controlled chamber. After the incubation, the sample was diluted in the 2 mL medium, filtered through 0.45 μm syringe filter, and titrated on Vero-TMPRSS2 cells. Virus titers were log-transformed to estimate a simple linear regression model. Virus titer at each time point was expressed as a geometric mean of three replicates and the standard deviation. A best-fit line and its 95% confidence interval of each regression model are represented by a solid line and its shaded area. The dashed line indicates the limit of detection where at least one sample out of the three replicates was positive by virus isolation and the empty symbols represent negative samples in all three replicates. On the *x* axis, 0.17 day are equal to 4 h.

In addition, the Omicron VOC was less stable than the WA-1 strain in liquid nasal mucus (*P* < 0.01) and medium (*P* < 0.01) under spring/fall conditions (Fig. 3). However, a significant difference was not observed in liquid sputum (Table 2).

**TABLE 2:** Half-life values of the SARS-CoV-2 Omicron variants of concern in liquid nasal mucus and sputum under spring/fall conditions

Biological fluid and virus strain	Spring/fall
	Liquid
Nasal mucus	
WA-1 lineage A	16.4 (11.93 to 26.2)^*a*^
Omicron VOC B.1.1.529	8.64 (6.1 to 14.81)
Sputum	
WA-1 lineage A	13.89 (10.09 to 22.3)
Omicron VOC B.1.1.529	13.89 (10.44 to 20.75)
Medium	
WA-1 lineage A	71.38 (45.37 to 167.24)
Omicron VOC B.1.1.529	30.32 (22.6 to 46.04)

a Half-life in hours (95% confidence interval).

## DISCUSSION

SARS-CoV-2 can be excreted in many types of biological fluids from infected individuals, but nasal mucus, sputum, and saliva are the primary components in the generation of respiratory droplets that play a major role in SARS-CoV-2 transmission (18). The infectious droplets can be inhaled directly, which is a primary route of SARS-CoV-2 transmission. In addition, very fine droplets can evaporate quickly in the air to create infectious aerosols, which can remain infectious for hours (13). In contrast, the larger droplets drop down to a nearby area within a few minutes and contaminate surfaces with infectious virus (see https://www.cdc.gov/coronavirus/2019-ncov/science/science-briefs/sars-cov-2-transmission.html). The virus that survives on surfaces can then be transferred by hand or other means to mucosal membranes in the oral or nasal cavity from contaminated surfaces (fomite transmission). Given this scenario, SARS-CoV-2 survival is of concern for its potential role in fomite transmission; this has led to the extensive investigations on the effect of surfaces (13, 19, 20), substrates (15–17, 21), and environmental factors (12, 22, 23) on SARS-CoV-2 stability.

It has been considered that virus stability outside the host is dependent on the intrinsic nature of the virus, type of surface, surrounding substrate, and environmental conditions. Previously, we reported extended survival of the ancestral SARS-CoV-2 strain under winter conditions compared to other seasonal climates on various surfaces and biological fluids, and differential virus decay rates depending on the types of surfaces and the type of human biological fluid (15, 20). In this study, to elucidate the effect of virus strain on virus stability, we prepared virus stocks of four SARS-CoV-2 strains using identical cell culture conditions to eliminate other variables besides the genetics of the virus. SARS-CoV-2 has an approximately 30 kb RNA genome encapsulated by the nucleocapsid protein, and the RNA-nucleocapsid complex is surrounded by an envelope made of a lipid bilayer from host cells. The spike, envelope, and membrane proteins are embedded in this outer lipid membrane and determine the distinct shape and structure of SARS-CoV-2. It is reasonable to suggest that structural factors, such as structural proteins and lipid membrane, are primarily responsible for virus stability of individual SARS-CoV-2 strains. In this context, amino acid differences in the structural proteins may play a critical role in differential virus stability as described in this study.

A well-characterized SARS-CoV-2 mutation is the aspartic acid to glycine substitution at the position 614 of the spike protein (D614G). This substitution was found in early 2020 in Europe and quickly became dominant worldwide. Initial observations showed that patients infected with the D614G variant had higher viral loads which had a significant effect on SARS-CoV-2 infectivity and transmissibility (24). Further studies demonstrated that SARS-CoV-2 harboring the single D614G mutation exhibited increased competitive fitness, and rapid transmission in primary human respiratory cells and animal models (25). Subsequent cryo-EM analysis indicated that the D614G substitution abolishes an interprotomer salt bridge between D614 and K854 and/or an interprotomer hydrogen bond between D614 and T859 which results in structural flexibility of the spike protein, allowing the S1 subunit of the protomer to be easily disassociated from the S2 subunit of the adjacent protomer (26–28). The resulting destabilization of the S1-S2 interface triggers a conformational change of the receptor binding domain (RBD) of the spike protein, toward the “up state,” leading to increased ACE2 binding and enhanced infectivity. All VOCs used in this study harbor this potentially destabilizing D614G substitution within the spike protein which could explain the reduced stability compared to the ancestral strain containing the D614. Another common mutation is N501Y in the spike protein, which is located within the receptor binding motif. This substitution influences the ability to bind ACE2 and evade antibody neutralization; however, it does not result in notable structural changes (29–31). Furthermore, for the Alpha VOC, the A570D substitution in the S1 subdomain of the spike protein forms a new salt bridge with K854 in the RBD “up state” conformation or K964 in RBD “down state” conformation (30, 31), or it forms a new hydrogen bond with N856 (32). In contrast, the S982A substitution in the S2 subdomain of the spike protein causes the loss of a hydrogen bond with G545 (30) or T547 (32). In addition, the D1118H substitution in the S2 subdomain of the spike protein forms a symmetric histidine triad near the base of the spike, whereas the T716 substitution eliminates an intraprotomer hydrogen bond with Q1071 (32). For the Beta VOC, the E484K substitution in the S1 subdomain of the spike protein results in the elimination of a hydrogen bond with F490, and destabilizes the RBD structure with a high frequency of disordered RBDs (32). The overall architecture of the Omicron VOC spike is similar to those of other SARS-CoV-2 strains, but several of its substitutions introduce an interprotomer new hydrogen bond or salt bridge between N856K and D568/T572 and a hydrogen bond between N764K and T315 (33, 34). In addition, other mutations in the nucleocapsid and envelope proteins of the VOCs might have effects on virus stability, although their roles in the structural stability of the virus particle remains unclear. It is likely that all amino acid substitutions which stabilize or destabilize the structure of the spike protein, as well as potentially other structural proteins, synchronously affect the stability of SARS-CoV-2.

We found significant differences among the stability of SARS-CoV-2 strains in various liquid biological fluids and medium, whereas no difference was observed in dried medium and biological fluids on a stainless steel surface. In particular, the WA-1 strain and the Beta VOC were more stable than the Alpha VOC under indoor, summer, spring/fall conditions in human nasal mucus, and the WA-1 strain was more stable than both VOCs under all four conditions in human sputum. Furthermore, the WA-1 strain was more stable than the Omicron VOC in nasal mucus. These results might indicate that components such as certain enzymes present in the liquid biological fluids are responsible for the different virus decay rates observed, since the drying process on surfaces causes the inactivation of these components due to lack of water. It is plausible that an active component in biological fluids exerts its antiviral activity more effectively against VOCs that harbor structurally less stable spike proteins in their envelopes. Previous studies have indicated that the single D614G substitution increased the stability of SARS-CoV-2. Planta et al. showed that the G614 virus was more stable than the D614 virus in Dulbecco’s PBS at 33°C, 37°C, and 42°C (35). Another study demonstrated that the D614 virus in DMEM lost a considerable degree of infectivity with a 3-log reduction between 14 and 30 days at 4°C, whereas only a 1-log reduction was found with the G614 virus (36). Moreover, two recent studies showed that the Omicron VOC is more stable than the ancestral strain on surfaces (37, 38). In the present study, we prepared the inocula under identical conditions and mixed them with biologically relevant body fluids to calculate the virus decay rates over a period of time under different environmental conditions. In contrast, in previous studies, the inocula were prepared in virus transport medium (19, 37) or PBS following ultracentrifugation (38), and 2 or 5 μL of inoculum was placed on surfaces. These differences in methodology and data analysis might explain why our results indicate that the ancestral lineage A strain was more stable than the VOCs used this study. Furthermore, there was a biphasic decay pattern under winter conditions, suggesting the virus was a inactivated very rapidly in an evaporating droplet at early time points.

There are several limitations in the present study. First, we prepared the virus stocks from a Vero-TMPRSS2 cell line originating from a nonhuman primate. A previous study showed that influenza viruses sharing the same genetic background exhibited different virus decay rates in water based on the cell line in which they were cultivated, suggesting an influence of host/cell origin on virus stability (39). In addition, the samples were also titrated on Vero-TMPRSS2 cells given that these cells have been widely used to characterize and compare the infectivity and pathogenesis of novel variants of SARS-CoV-2. However, recent studies have shown that the variants utilize different viral entry pathway to cells; thus, it cannot be ruled out that differential infectivity in Vero-TMPRSS2 cells could potentially affect the data obtained in this study (40). Second, we used early isolates of Alpha and Beta VOCs (isolated in November 2020) and the Omicron VOC (isolated in November 2021). Even though SARS-CoV-2 strains are classified into defined VOCs, some VOC isolates have accumulated additional mutations in their structural proteins over time, resulting in the emergence of novel sublineages within the same VOC. Importantly, Omicron BA.1 used in this study, circulated for a relatively short period and was quickly replaced by the BA.2 subvariant, followed by the BA.5 subvariant. It is possible that the different mutations in the spike protein of these more recent Omicron subvariants might have a different effect on their stability compared to the BA.1 isolate described here. In addition, we found spontaneous amino acid substitutions that emerged after serial passages in cell culture, such as Q677H and R682W in the spike protein of the Beta VOC. Amino acid substitutions from additional or spontaneous mutations may also impact virus stability.

The main route of SARS-CoV-2 transmission occurs when an individual directly inhales respiratory droplets or aerosols from a nearby infected person. On the other hand, fomite transmission seems to play a lesser role in SARS-CoV-2 transmission. Through the evolutionary processes, SARS-CoV-2 has acquired the ability to adapt and survive in various hostile environments; it was able to evade pre-existing immunity and to evolve to more efficient transmissibility. Given this scenario, it is plausible that less stable VOCs became dominant worldwide because such variants seem to show enhanced transmissibility and increased fitness in mammals. In conclusion, our data indicate that fomite transmission of SARS-CoV-2 VOCs seems less likely via liquid nasal mucus and sputum. In contrast, in dried biological fluids, SARS-CoV-2 VOCs more than the ancestral strains could still pose a significant fomite transmission risk. The present work provides novel insights into the stability of SARS-CoV-2 and its VOCs and could, therefore, contribute to the development of mitigation strategies to reduce fomite transmission.

## MATERIALS AND METHODS

### Cell and virus.

Vero-TMPRRS2 cells were cultured in Dulbecco’s modified Eagle Medium (DMEM; Corning, Manassas, VA, USA) supplemented with 10% fetal bovine serum (FBS; R&D systems, Flower Branch, GA, USA), 1% antibiotic-antimycotic solutions (Gibco, Grand Island, NY, USA), and Geneticin (Gibco, Grand Island, NY, USA) and maintained at 37°C in a humidified 5% CO2 incubator. In this study, we used four different SARS-CoV-2 strains: (i) USA-WA/2020 which was isolated from the first U.S. patient in January 2020 (BEI catalog number: NR-52281; herein as WA-1); (ii) hCoV-19/England/204820464/2020 which was isolated in November 2020 in United Kingdom (NR-54000; herein as Alpha VOC); (iii) hCoV-19/South Africa/KRISP-K005325/2020 which was isolated in November 2020 in South Africa (NR-54009; herein as Beta VOC); and (iv) hCoV-19/USA/NY-MSHSPSP-PV44476/2021 which was isolated in November 2021 in New York, USA (herein as Omicron VOC). Virus stocks were prepared in Vero-TMPRRS2 cells which were maintained in virus growth medium (DMEM supplemented with 5% FBS and 1% antibiotic-antimycotic solutions). The titer of virus stocks was determined using endpoint titration in Vero-TMPRRS2 cells.

### Next-generation sequencing.

Viral RNA was extracted from virus stocks using a magnetic bead based automatic extraction system (Taco DNA/RNA Extraction Kit, GeneReach, Lexington, MA, USA) and sequenced by next-generation sequencing using an Illumina NextSeq (Illumina, Inc., San Diego, CA, USA) as reported previously (41–43). Briefly, nucleic acid extractions were performed by combining equal amounts of cell culture supernatants with RLT Lysis Buffer (Qiagen, Germantown, MA, USA), with 200 μL of the lysate used for magnetic bead-based extraction according to the manufacturer’s protocol. SARS-CoV-2 cDNA was then synthesized and amplified using the ARTIC-V3 RT-PCR protocol reference: Josh Quick 2020. nCOV-2019 sequencing protocol versus (GunIt), https://doi.org/10.17504/protocols.io.bdp7i5rn, followed by library preparation using a Nextera XT library prep kit (Illumina, Inc., San Diego, CA, USA) according to manufacturer’s protocols. The libraries were then sequenced with the Illumina NextSeq platform using paired-end 150 bp reads. Reads were then demultiplexed and parsed into individual sample files that were imported into CLC Genomics Workbench version 7.5 (Qiagen, Germantown, MD, USA) for analysis. Reads were trimmed to remove ambiguous nucleotides at the 5′ end and filtered to remove low quality and short reads. To determine amino acid substitutions in the Alpha and Beta VOCs, sequencing reads were mapped to the WA-1 (GISAID accession ID: EPI_ISL_404895) consensus sequence, followed by analysis with the low frequency variant detector program in CLC Genomics workbench to determine nonsynonymous substitutions. The consensus sequences for each of the variant isolates were extracted from the read mappings. Consensus sequences were then aligned with published sequences from the GISAID database (GISAID accession ID: EPI_ISL_683466 for the Alpha VOC, EPI_ISL_678615 for the Beta VOC, and EPI_ISL_7908052 for Omicron VOC) to manually inspect all identified mutations. The undetermined regions in next-generation sequencing were further confirmed by Sanger sequencing using primers in the Midnight protocol (https://doi.org/10.17504/protocols.io.bwyppfvn).

### Virus stability assay.

To prevent potential cross-contamination during the work, the stability assay for each virus strain was performed at separate times, and surfaces of the biosafety cabinet and equipment were thoroughly decontaminated between work with different strains using appropriate disinfectant. In the first study, inoculums of WA-1, Alpha VOC, and Beta VOC were prepared by diluting the virus stock in virus growth medium at the concentration of 10^7^ TCID_50_/mL. A total 5 μL of the diluted virus (5 × 10^4^ TCID_50_) was directly mixed with 0.1 g to 0.2 g of human nasal mucus or sputum (Lee Biosolutions, Inc., Maryland Heights, MO, USA) in a 2 mL tube or on a stainless steel surface in a 12-well plate. The same amount of the virus was mixed with 45 μL of human saliva (Lee Biosolutions) or medium and transferred into a 2 mL tube or onto stainless steel in the 12-well plate. The mixture on the steel surface was completely air-dried in a biosafety cabinet for 4 h. The virus-spiked biologicals in the 2 mL tube and on stainless steel were then placed in a temperature- and humidity-controlled chamber (Nor-Lake Scientific, Hudson, WI, USA) under four different environmental conditions: 21°C/60% relative humidity (RH), 25°C/70% RH, 13°C/66% RH, and 5°C/75% RH. These conditions simulated the indoor, summer, spring/fall, and winter climatic conditions for the Midwestern United States (15, 20). Three virus-spiked biological samples per each time point (Table S2) were subject to virus isolation in Vero-TMPRSS2 cells. Briefly, the infectious virus was recovered in 2 mL of the virus growth medium, vortexed thoroughly, and filtered through a 0.45 μm syringe filter. Ten-fold serial dilutions were prepared and transferred onto Vero-TMPRSS2 cells in a 96-well plate. The presence of cytopathic effect was recorded at 4 days, and the virus titer was calculated using Reed-Muench method.

The second study was carried out to determine the stability of Omicron VOC in liquid nasal mucus and sputum. The inoculums of WA-1 and Omicron VOC were prepared in virus growth medium at the concentration of 6.2 × 10^6^ TCID_50_/mL, and 5 μL of the diluted virus (3.1 × 10^4^ TCID_50_) was directly mixed with 0.1 g to 0.2 g of human nasal mucus or sputum. The mixtures were incubated only under spring/fall conditions for which the different stability of Alpha and Beta VOCs were clearly demonstrated. Samples at each time points were processed as mentioned above.

The log-transformed virus titers from the first time point (1.5 h or 4 h) to the last positive time point when at least one out of three replicates was positive were used to estimate a simple linear regression using GraphPad Prism 9 software (GraphPad, San Diego, CA, USA). The half-life value was calculated as − log_10_ (2)/slope. Statistical difference in half-life values between WA-1, Alpha VOC, and Beta VOC were evaluated by one-way analysis of variance (ANOVA), followed by multiple pairwise comparisons using Tukey’s adjustment according to the software’s instruction. In addition, statistical difference between WA-1 and Omicron VOC was tested using default analysis, which is compatible to analysis of covariance in GraphPad Prism 9. The virus decay in nasal mucus and sputum are shown in figures to illustrate the significant differences observed, and all half-life values are presented in the tables.

## References

[1] Bhatt PR, Scaiola A, Loughran G, Leibundgut M, Kratzel A, Meurs R, Dreos R, O'Connor KM, McMillan A, Bode JW, Thiel V, Gatfield D, Atkins JF, Ban NN. 2021. Structural basis of ribosomal frameshifting during translation of the SARS-CoV-2 RNA genome. Science 372:1306–1313. doi:10.1126/science.abf3546. 34029205 PMC8168617

[2] Suryawanshi RK, Koganti R, Agelidis A, Patil CD, Shukla D. 2021. Dysregulation of cell signaling by SARS-CoV-2. Trends Microbiol 29:224–237. doi:10.1016/j.tim.2020.12.007. 33451855 PMC7836829

[3] Cai YF, Zhang J, Xiao TS, Peng HQ, Sterling SM, Walsh RM, Rawson S, Rits-Volloch S, Chen B. 2020. Distinct conformational states of SARS-CoV-2 spike protein. Science 369:1586–1592. doi:10.1126/science.abd4251. 32694201 PMC7464562

[4] Boson B, Legros V, Zhou B, Siret E, Mathieu C, Cosset F-L, Lavillette D, Denolly S, Denolly S. 2021. The SARS-CoV-2 envelope and membrane proteins modulate maturation and retention of the spike protein, allowing assembly of virus-like particles. J Biol Chem 296:100111. doi:10.1074/jbc.RA120.016175. 33229438 PMC7833635

[5] Schoeman D, Fielding BC. 2019. Coronavirus envelope protein: current knowledge. Virol J 16:69. doi:10.1186/s12985-019-1182-0. 31133031 PMC6537279

[6] Neuman BW, Kiss G, Kunding AH, Bhella D, Baksh MF, Connelly S, Droese B, Klaus JP, Makino S, Sawicki SG, Siddell SG, Stamou DG, Wilson IA, Kuhn P, Buchmeier MJ. 2011. A structural analysis of M protein in coronavirus assembly and morphology. J Struct Biol 174:11–22. doi:10.1016/j.jsb.2010.11.021. 21130884 PMC4486061

[7] Cubuk J, Alston JJ, Incicco JJ, Singh S, Stuchell-Brereton MD, Ward MD, Zimmerman MI, Vithani N, Griffith D, Wagoner JA, Bowman GR, Hall KB, Soranno A, Holehouse AS. 2021. The SARS-CoV-2 nucleocapsid protein is dynamic, disordered, and phase separates with RNA. Nat Commun 12:1936. doi:10.1038/s41467-021-21953-3. 33782395 PMC8007728

[8] Davies NG, Abbott S, Barnard RC, Jarvis CI, Kucharski AJ, Munday JD, Pearson CAB, Russell TW, Tully DC, Washburne AD, Wenseleers T, Gimma A, Waites W, Wong KLM, van Zandvoort K, Silverman JD, Diaz-Ordaz K, Keogh R, Eggo RM, Funk S, Jit M, Atkins KE, Edmunds WJ, CMMID COVID-19 Working Group. 2021. Estimated transmissibility and impact of SARS-CoV-2 lineage B.1.1.7 in England. Science 372:eabg3055. doi:10.1126/science.abg3055. 33658326 PMC8128288

[9] Wang PF, Casner RG, Nair MS, Wang M, Yu J, Cerutti G, Liu LH, Kwong PD, Huang YX, Shapiro L, Ho DD. 2021. Increased resistance of SARS-CoV-2 variant P.1 to antibody neutralization. Cell Host Microbe 29:747–751.e4. doi:10.1016/j.chom.2021.04.007. 33887205 PMC8053237

[10] Wang PF, Nair MS, Liu LH, Iketani S, Luo Y, Guo YC, Wang M, Yu J, Zhang BS, Kwong PD, Graham BS, Mascola JR, Chang JY, Yin MT, Sobieszczyk M, Kyratsous CA, Shapiro L, Sheng ZZ, Huang YX, Ho DD. 2021. Antibody resistance of SARS-CoV-2 variants B.1.351 and B.1.1.7. Nature 593:130–135. doi:10.1038/s41586-021-03398-2. 33684923

[11] Argimon S, Abudahab K, Goater RJE, Fedosejev A, Bhai J, Glasner C, Feil EJ, Holden MTG, Yeats CA, Grundmann H, Spratt BG, Aanensen DM. 2016. Microreact: visualizing and sharing data for genomic epidemiology and phylogeography. Microb Genom 2. 10.1099/mgen.0.000093 PMC5320705 28348833

[12] Biryukov J, Boydston JA, Dunning RA, Yeager JJ, Wood S, Reese AL, Ferris A, Miller D, Weaver W, Zeitouni NE, Phillips A, Freeburger D, Hooper I, Ratnesar-Shumate S, Yolitz J, Krause M, Williams G, Dawson DG, Herzog A, Dabisch P, Wahl V, Hevey MC, Altamura LA. 2020. Increasing temperature and relative humidity accelerates inactivation of SARS-CoV-2 on surfaces. mSphere 5:e00441-20. doi:10.1128/mSphere.00441-20. 32611701 PMC7333574

[13] van Doremalen N, Bushmaker T, Morris DH, Holbrook MG, Gamble A, Williamson BN, Tamin A, Harcourt JL, Thornburg NJ, Gerber SI, Lloyd-Smith JO, de Wit E, Munster VJ. 2020. Aerosol and surface stability of SARS-CoV-2 as compared with SARS-CoV-1. N Engl J Med 382:1564–1567. doi:10.1056/NEJMc2004973. 32182409 PMC7121658

[14] Todt D, Meister TL, Tamele B, Howes J, Paulmann D, Becker B, Brill FH, Wind M, Schijven J, Heinen N, Kinast V, Mhlekude B, Goffinet C, Krawczyk A, Steinmann J, Pfaender S, Bruggemann Y, Steinmann E. 2021. A realistic transfer method reveals low risk of SARS-CoV-2 transmission via contaminated euro coins and banknotes. iScience 24:102908. doi:10.1016/j.isci.2021.102908. 34337354 PMC8312053

[15] Kwon T, Gaudreault NN, Richt JA. 2021. Seasonal stability of SARS-CoV-2 in biological fluids. Pathogens 10:540. doi:10.3390/pathogens10050540. 33946190 PMC8147080

[16] Matson MJ, Yinda CK, Seifert SN, Bushmaker T, Fischer RJ, van Doremalen N, Lloyd-Smith JO, Munster VJ. 2020. Effect of environmental conditions on SARS-CoV-2 stability in human nasal mucus and sputum. Emerg Infect Dis 26:2276–2278. doi:10.3201/eid2609.202267. 32511089 PMC7454058

[17] Liu Y, Li T, Deng Y, Liu S, Zhang D, Li H, Wang X, Jia L, Han J, Bei Z, Li L, Li J. 2021. Stability of SARS-CoV-2 on environmental surfaces and in human excreta. J Hosp Infect 107:105–107. doi:10.1016/j.jhin.2020.10.021. 33137445 PMC7603996

[18] Atkinson J, Chartier Y, Pessoa-Silva CL, Jensen P, Li Y, Seto WH. 2009. Natural ventilation for infection control in health-care settings [review]. World Health Organization, Geneva, Switzerland. 23762969

[19] Chin AWH, Chu JTS, Perera MRA, Hui KPY, Yen H-L, Chan MCW, Peiris M, Poon LLM. 2020. Stability of SARS-CoV-2 in different environmental conditions. Lancet Microbe 1:e10. doi:10.1016/S2666-5247(20)30003-3. 32835322 PMC7214863

[20] Kwon T, Gaudreault NN, Richt JA. 2021. Environmental stability of SARS-CoV-2 on different types of surfaces under indoor and seasonal climate conditions. Pathogens 10:227. doi:10.3390/pathogens10020227. 33670736 PMC7922895

[21] Pastorino B, Touret F, Gilles M, de Lamballerie X, Charrel RN. 2020. Prolonged infectivity of SARS-CoV-2 in fomites. Emerg Infect Dis 26:2256–2257. doi:10.3201/eid2609.201788. 32579874 PMC7454106

[22] Ratnesar-Shumate S, Williams G, Green B, Krause M, Holland B, Wood S, Bohannon J, Boydston J, Freeburger D, Hooper I, Beck K, Yeager J, Altamura LA, Biryukov J, Yolitz J, Schuit M, Wahl V, Hevey M, Dabisch P. 2020. Simulated sunlight rapidly inactivates SARS-CoV-2 on surfaces. J Infect Dis 222:214–222. doi:10.1093/infdis/jiaa274. 32432672 PMC7313905

[23] Riddell S, Goldie S, Hill A, Eagles D, Drew TW. 2020. The effect of temperature on persistence of SARS-CoV-2 on common surfaces. Virol J 17:145. doi:10.1186/s12985-020-01418-7. 33028356 PMC7538848

[24] Volz E, Hill V, McCrone JT, Price A, Jorgensen D, O'Toole A, Southgate J, Johnson R, Jackson B, Nascimento FF, Rey SM, Nicholls SM, Colquhoun RM, da Silva Filipe A, Shepherd J, Pascall DJ, Shah R, Jesudason N, Li K, Jarrett R, Pacchiarini N, Bull M, Geidelberg L, Siveroni I, Consortium C-U, Goodfellow I, Loman NJ, Pybus OG, Robertson DL, Thomson EC, Rambaut A, Connor TR, COG-UK Consortium. 2021. Evaluating the effects of SARS-CoV-2 spike mutation D614G on transmissibility and pathogenicity. Cell 184:64–75.e11. doi:10.1016/j.cell.2020.11.020. 33275900 PMC7674007

[25] Hou YXJ, Chiba S, Halfmann P, Ehre C, Kuroda M, Dinnon KH, Leist SR, Schafer A, Nakajima N, Takahashi K, Lee RE, Mascenik TM, Graham R, Edwards CE, Tse LV, Okuda K, Markmann AJ, Bartelt L, de Silva A, Margolis DM, Boucher RC, Randell SH, Suzuki T, Gralinski LE, Kawaoka Y, Baric RS. 2020. SARS-CoV-2 D614G variant exhibits efficient replication ex vivo and transmission in vivo. Science 370:1464–1468. doi:10.1126/science.abe8499. 33184236 PMC7775736

[26] Zhang J, Cai Y, Xiao T, Lu J, Peng H, Sterling SM, Walsh RM Jr., Rits-Volloch S, Zhu H, Woosley AN, Yang W, Sliz P, Chen B. 2021. Structural impact on SARS-CoV-2 spike protein by D614G substitution. Science 372:525–530. doi:10.1126/science.abf2303. 33727252 PMC8139424

[27] Yurkovetskiy L, Wang X, Pascal KE, Tomkins-Tinch C, Nyalile TP, Wang Y, Baum A, Diehl WE, Dauphin A, Carbone C, Veinotte K, Egri SB, Schaffner SF, Lemieux JE, Munro JB, Rafique A, Barve A, Sabeti PC, Kyratsous CA, Dudkina NV, Shen K, Luban J. 2020. Structural and functional analysis of the D614G SARS-CoV-2 spike protein variant. Cell 183:739–751.e8. doi:10.1016/j.cell.2020.09.032. 32991842 PMC7492024

[28] Ozono S, Zhang Y, Ode H, Sano K, Tan TS, Imai K, Miyoshi K, Kishigami S, Ueno T, Iwatani Y, Suzuki T, Tokunaga K. 2021. SARS-CoV-2 D614G spike mutation increases entry efficiency with enhanced ACE2-binding affinity. Nat Commun 12:848. doi:10.1038/s41467-021-21118-2. 33558493 PMC7870668

[29] Zhu X, Mannar D, Srivastava SS, Berezuk AM, Demers JP, Saville JW, Leopold K, Li W, Dimitrov DS, Tuttle KS, Zhou S, Chittori S, Subramaniam S. 2021. Cryo-electron microscopy structures of the N501Y SARS-CoV-2 spike protein in complex with ACE2 and 2 potent neutralizing antibodies. PLoS Biol 19:e3001237. doi:10.1371/journal.pbio.3001237. 33914735 PMC8112707

[30] Cai Y, Zhang J, Xiao T, Lavine CL, Rawson S, Peng H, Zhu H, Anand K, Tong P, Gautam A, Lu S, Sterling SM, Walsh RM Jr., Rits-Volloch S, Lu J, Wesemann DR, Yang W, Seaman MS, Chen B. 2021. Structural basis for enhanced infectivity and immune evasion of SARS-CoV-2 variants. Science 373:642–648. doi:10.1126/science.abi9745. 34168070 PMC9245151

[31] Yang TJ, Yu PY, Chang YC, Liang KH, Tso HC, Ho MR, Chen WY, Lin HT, Wu HC, Hsu SD. 2021. Effect of SARS-CoV-2 B.1.1.7 mutations on spike protein structure and function. Nat Struct Mol Biol 28:731–739. doi:10.1038/s41594-021-00652-z. 34385690

[32] Gobeil SM, Janowska K, McDowell S, Mansouri K, Parks R, Stalls V, Kopp MF, Manne K, Li D, Wiehe K, Saunders KO, Edwards RJ, Korber B, Haynes BF, Henderson R, Acharya P. 2021. Effect of natural mutations of SARS-CoV-2 on spike structure, conformation, and antigenicity. Science 373:eabi6226. doi:10.1126/science.abi6226. 34168071 PMC8611377

[33] Gobeil SM, Henderson R, Stalls V, Janowska K, Huang X, May A, Speakman M, Beaudoin E, Manne K, Li D, Parks R, Barr M, Deyton M, Martin M, Mansouri K, Edwards RJ, Eaton A, Montefiori DC, Sempowski GD, Saunders KO, Wiehe K, Williams W, Korber B, Haynes BF, Acharya P. 2022. Structural diversity of the SARS-CoV-2 Omicron spike. Mol Cell 82:2050–2068.e6. doi:10.1016/j.molcel.2022.03.028. 35447081 PMC8947964

[34] Zhang J, Cai Y, Lavine CL, Peng H, Zhu H, Anand K, Tong P, Gautam A, Mayer ML, Rits-Volloch S, Wang S, Sliz P, Wesemann DR, Yang W, Seaman MS, Lu J, Xiao T, Chen B. 2022. Structural and functional impact by SARS-CoV-2 Omicron spike mutations. Cell Rep 39:110729. doi:10.1016/j.celrep.2022.110729. 35452593 PMC8995406

[35] Plante JA, Liu Y, Liu JY, Xia HJ, Johnson BA, Lokugamage KG, Zhang XW, Muruato AE, Zou J, Fontes-Garfias CR, Mirchandani D, Scharton D, Bilello JP, Ku ZQ, An ZQ, Kalveram B, Freiberg AN, Menachery VD, Xie XP, Plante KS, Weaver SC, Shi PY. 2021. Spike mutation D614G alters SARS-CoV-2 fitness. Nature 592:116–121. doi:10.1038/s41586-020-2895-3. 33106671 PMC8158177

[36] Huang SY, Kung YA, Huang PN, Chang SY, Gong YN, Han YJ, Chiang HJ, Liu KT, Lee KM, Chang CY, Chang CC, Huang CG, Shih SR. 2021. Stability of SARS-CoV-2 Spike G614 variant surpasses that of the D614 variant after cold storage. Msphere 6:e00104-21. doi:10.1128/mSphere.00104-21. 33789940 PMC8546686

[37] Chin AWH, Lai AMY, Peiris M, Man Poon LL. 2022. Increased stability of SARS-CoV-2 Omicron variant over ancestral strain. Emerg Infect Dis 28:1515–1517. doi:10.3201/eid2807.220428. 35550234 PMC9239870

[38] Hirose R, Itoh Y, Ikegaya H, Miyazaki H, Watanabe N, Yoshida T, Bandou R, Daidoji T, Nakaya T. 2022. Differences in environmental stability among SARS-CoV-2 variants of concern: both Omicron BA.1 and BA.2 have higher stability. Clin Microbiol Infect In press. 10.1016/j.cmi.2022.05.020 PMC9144845 35640841

[39] Shigematsu S, Dublineau A, Sawoo O, Batejat C, Matsuyama T, Leclercq I, Manuguerra JC. 2014. Influenza A virus survival in water is influenced by the origin species of the host cell. Influenza Other Respir Viruses 8:123–130. doi:10.1111/irv.12179. 24112132 PMC4177806

[40] Fan Y, Li X, Zhang L, Wan S, Zhang L, Zhou F. 2022. SARS-CoV-2 Omicron variant: recent progress and future perspectives. Signal Transduct Target Ther 7:141. doi:10.1038/s41392-022-00997-x. 35484110 PMC9047469

[41] Gaudreault NN, Trujillo JD, Carossino M, Meekins DA, Morozov I, Madden DW, Indran SV, Bold D, Balaraman V, Kwon T, Artiaga BL, Cool K, Garcia-Sastre A, Ma W, Wilson WC, Henningson J, Balasuriya UBR, Richt JA. 2020. SARS-CoV-2 infection, disease and transmission in domestic cats. Emerg Microbes Infect 9:2322–2332. doi:10.1080/22221751.2020.1833687. 33028154 PMC7594869

[42] Meekins DA, Morozov I, Trujillo JD, Gaudreault NN, Bold D, Carossino M, Artiaga BL, Indran SV, Kwon T, Balaraman V, Madden DW, Feldmann H, Henningson J, Ma WJ, Balasuriya UBR, Richt JA. 2020. Susceptibility of swine cells and domestic pigs to SARS-CoV-2. Emerg Microbes Infect 9:2278–2288. doi:10.1080/22221751.2020.1831405. 33003988 PMC7594707

[43] Cool K, Gaudreault NN, Morozov I, Trujillo JD, Meekins DA, McDowell C, Carossino M, Bold D, Mitzel D, Kwon T, Balaraman V, Madden DW, Artiaga BL, Pogranichniy RM, Roman-Sosa G, Henningson J, Wilson WC, Balasuriya UBR, Garcia-Sastre A, Richt JA. 2022. Infection and transmission of ancestral SARS-CoV-2 and its alpha variant in pregnant white-tailed deer. Emerg Microbes Infect 11:95–112. doi:10.1080/22221751.2021.2012528. 34842046 PMC8725908

