# Supplementary material for: Ancestral Lineage of SARS-CoV-2 Is More Stable in Human Biological Fluids than Alpha, Beta, and Omicron Variants of Concern
Source: Microbiol Spectr. 2023 Jan 23;11(1):e03301-22. doi: 10.1128/spectrum.03301-22 (PMC9927102; doi:10.1128/spectrum.03301-22)
Supplement: Supplemental file 1 — Tables S1 and S2. Download spectrum.03301-22-s0001.pdf, PDF file, 0.1 MB [file spectrum.03301-22-s0001.pdf]

Supplementary Table 1. Comparison of consensus amino acid sequences among virus stocks used in this study.

Viral protein	Amino acid position	WA-1 lineage A	Alpha VOC B.1.1.7	Beta VOC B.1.351	Omicron VOC B.1.1.529
Spike	18	L	L	F	L
	67	A	A	A	V
	69–70	HV	Deletion	HV	Deletion
	80	D	D	A	D
	95	T	T	T	I
	142	G	G	G	D
	143–144	VY	VY	VY	Deletion
	145	Y	Deletion	Y	Deletion
	211	N	N	N	Deletion
	212	L	L	L	I
	214				EPE (Insertion)
	215	D	D	G	D
	242–244	LAL	LAL	Deletion	LAL
	339	G	G	G	D
	371	S	S	S	L
	373	S	S	S	P
	375	S	S	S	F
	417	K	K	N	N
	440	N	N	N	K
	446	G	G	G	S
	477–478	ST	ST	ST	NK
	484	E	E	K	A
	493	Q	Q	Q	R
	496	G	G	G	S
	498	Q	Q	Q	R
	501	N	Y	Y	Y
	505	Y	Y	Y	H
	547	T	T	T	K
	570	A	D	A	A
	614	D	G	G	G
	655	H	H	H	Y
	677	Q	Q	H (Q in the reference sequence) [a]	Q
	679	N	N	N	K
	681	P	H	P	H
	682	R	R	W (R in the reference sequence) [a]	R
	701	A	A	V	V
	716	T	I	T	T
	764	N	N	N	K
	796	D	D	D	Y
	856	N	N	N	K
	954	Q	Q	Q	H
	969	N	N	N	K
	981	L	L	L	F
	982	S	A	S	S
	1118	D	H	D	D

Envelope	9	T	T	T	I
	71	P	P	L	P
Matrix	3	D	D	D	G
	19	Q	Q	Q	E
	63	A	A	A	T
Nucleocapsid	3	D	L	D	D
	13	P	P	P	L
	31–33	ERS	ERS	ERS	Deletion
	203–204	RG	KR	RG	KR
	205	T	T	I	T
	235	S	F	S	S
NSP2	85	T	T	I	T
NSP3	38	K	K	K	R
	183	T	I	T	T
	837	K	K	N	K
	890	A	D	A	A
	1069	V	V	V	I
	1265	S	S	S	Deletion
	1266	L	L	L	I
	1305	A	V	A	A
	1412	I	T	I	I
	1892	A	A	A	T
NSP4	492	T	T	T	I
NSP5	90	K	K	R	K
	132	P	P	P	H
	252	P	P	L (P in the reference sequence) [a]	P
NSP6	105	L	L	L	Deletion
	106	S	Deletion	Deletion	Deletion
	107	G	Deletion	Deletion	Deletion
	108	F	Deletion	Deletion	F
	189	I	I	I	V
NSP12	323	P	L	L	L
NSP13	460	K	R	K	K
NSP14	42	I	I	I	V
	347	E	G	E	E
ORF3a	57	Q	Q	H	Q
	131	W	W	L	W
	171	S	S	L	S
ORF7a	93	V	V	F	V
ORF8	27	Q	Stop codon	Q	Q
	84	S	Not determined	L	L
	115	R	Not determined	L (R in the reference sequence) [a]	R

a Spontaneous mutation which was found in our virus stock, when compared to the reference sequence in GISAID (EPI_ISL_678615).

Supplementary Table 2. Timeline for sample collections after contamination under environmental conditions.

	21 °C/60% RH, indoor,<br>25 °C/70% RH, summer	13 °C/66% RH, spring/fall	5 °C/75% RH, winter
Time points	4 hours, 1, 2, 3, and 4 days [a]	4 hours, 1, 3, 5, and 7 days	4 hours, 1, 3, 6, 10, 16, and 21 days

[a] For liquid nasal mucus, liquid sputum and dried nasal mucus, time points were 1.5, 4, 8, 24, and 48 hours post-contamination.
